# Economics: The Clear Advantage of Clean Air

**DOI:** 10.1289/ehp.114-a154a

**Published:** 2006-03

**Authors:** Tim Lougheed

Regulations addressing environmental problems such as air pollution might be dismissed as a luxury for rich countries that can afford it, but members of the UN Environment Programme (UNEP) insist that such measures actually represent the most effective means of lifting countries out of poverty. At a major gathering of environment ministers in the United Arab Emirates in February 2006, UNEP executive director Klaus Töpfer cited the increasing cost and demand for fossil fuels as key reasons why many rapidly developing countries were taking a closer look at environmental degradation, which can have profound consequences. “That is now the bottleneck to future economic development,” he said.

The extent of the economic implications of this bottleneck is being outlined by researchers at the Massachusetts Institute of Technology (MIT), who have expanded a well-established economic model to put some numbers to the economic disadvantages created by unregulated air pollution. Those numbers are quite big, as it turns out. In a July 2004 report titled *Economic Benefits of Air Pollution Regulation in the USA: An Integrated Approach*, they calculated that the United States enjoyed an additional $5.4 trillion in market consumption between 1975 and 2000 that would not have been available without the implementation of air pollution controls.

For coauthor John Reilly, associate director of research with MIT’s Joint Program on the Science and Policy of Global Change and the Laboratory for Energy and the Environment, that amount dwarfs the less than $1 trillion that the EPA estimates the controls cost. More importantly, this financial benefit contrasts sharply with the widespread health effects of leaving remaining air pollution unregulated. “It’s causing your workers to be ill, and your children not to be able to advance,” he argues. “It’s going to slow the economy.”

Using the Emissions Prediction and Policy Analysis (EPPA) model, which has already been used to estimate the costs of reducing carbon dioxide emissions, Reilly and his colleagues incorporated measures of human health in order to consider the benefits. Rather than employing only basic multipliers for illness, lost lives, or medical expenditures, the algorithms were broadened with published health data on the occurrence of specific diseases that could be linked to air pollution, such as asthma.

By including pollutant exposures, ability to work, and health effects on different age groups over time, the model compared actual economic performance with what would have happened in the absence of regulations. The tally wound up representing 3–4% of the entire U.S. market consumption over a quarter of a century. By comparison, the entire agricultural sector accounts for just 1%.

That result should grab the attention of observers in developed as well as developing countries, says Kristie Ebi, an epidemiologist with the scientific consultancy Exponent Health Group. “Health is often not at the table,” she says, referring to the negotiation of policies that treat the physical well-being of people as a separate priority incurring expenses that are not considered part of the general economy. “Many calculations of the costs and benefits of regulations include limited consideration of health because health is not traded in a market and so is hard to value.”

For Ebi, the expanded EPPA model provides all the more reason to make health an integral part of such negotiations and look closely at the potential advantages conferred by environmental regulations. “There’s very little work out there that you can take to policy makers and say ‘if you don’t regulate, this is the cost,’” she concludes. “It’s really nice to see that taken up in a rigorous way.”

